# Exploring Endogenous and Exogenous Factors for Successful Artificial Insemination in Sheep: A Global Overview

**DOI:** 10.3390/vetsci11020086

**Published:** 2024-02-11

**Authors:** Bouchra El Amiri, Abdellatif Rahim

**Affiliations:** 1 Animal Production Unit, Regional Center Agricultural Research of Settat, National Institute for Agricultural Research (INRA), Avenue Ennasr, P.O. Box 415 Rabat Principal, Rabat 10090, Morocco; a.rahim@uhp.ac.ma; 2 African Sustainable Agriculture Research Institute (ASARI), Mohammed VI Polytechnic University (UM6P), Laayoune 70000, Morocco; 3 Laboratory of Biochemistry, Neurosciences, Natural Resources and Environment, Faculty of Sciences and Techniques, Hassan First University of Settat, P.O. Box 577, Settat 26000, Morocco

**Keywords:** artificial insemination, sheep, endogenous factors, exogenous factors, breeding success

## Abstract

**Simple Summary:**

In this overview of the literature, we explore the complex endogenous and exogenous factors influencing the success of ovine artificial insemination (AI). From genetic dynamics and age-related fertility variations to the complexities of cervical anatomy and semen quality, we explore the nuances impacting ovine AI. Additionally, we examine the ongoing debate between natural and hormonal synchronization, semen handling, and the profound effects of environmental stressors like heat and nutritional stress. Our findings suggest that a holistic approach, considering both endogenous and exogenous factors, is crucial for optimizing AI outcomes in sheep breeding programs. This overview of the literature serves as a valuable guide for researchers, veterinarians, and sheep breeders, offering practical insights to enhance the efficiency and success of ovine AI programs and contribute to improved reproductive outcomes and genetic progress.

**Abstract:**

Artificial insemination (AI) plays a vital role in animal breeding programs. AI is applied to enhance animal genetics and facilitate the widespread integration of desirable characteristics with a high potential for productivity. However, in sheep, this biotechnology is not commonly practicable due to multi-factorial challenges, resulting in inconsistent outcomes and unpredictable results. Thoughtful selection of semen donors and recipients based on genetic merit deeply impacts ovine AI outcomes. Additionally, endogenous factors such as breed, age, fertility traits, genetic disorders, and cervical anatomy in ewes contribute to ovine AI success. Extensive research has studied exogenous influences on sexual behavior, reproductive health, and hormonal regulation, all impacting ovine AI success. These exogenous factors include techniques like estrus induction, synchronization, semen handling methods (fresh/chilled/frozen), and insemination methods (cervical/laparoscopic), as well as nutritional factors and climatic conditions. This overview of the literature highlights the endogenous and exogenous challenges facing successful ovine AI and proposes strategies and best practices for improvement. This paper will serve as a guide for understanding and optimizing the success of ovine AI.

## 1. Introduction

Artificial insemination (AI) plays a key role in modern animal breeding programs [1]. It offers a valuable combination of economic benefits and precise genetic control, making it a fundamental strategy for enhancing the genetics and productivity of animal populations [2]. Furthermore, it has highly transformed and impacted the management of animal reproduction, enabling the efficient propagation of desirable traits and overcoming geographical constraints in animal breeding [3]. However, in sheep, AI is not globally adopted due to the challenges associated with irregular and low fertility results [4]. Sheep are associated with several endogenous and exogenous factors that can vary significantly between individuals and breeds, affecting the effectiveness of AI [5,6]. The impact of exogenous factors, such as estrus induction, synchronization [7,8], semen collection methods [9], and choices between fresh, chilled, or frozen semen [10], has been the subject of extensive studies in the context of ovine AI. However, it is equally important to recognize the impact of endogenous factors to the success of ovine AI programs. The careful selection of semen donors and recipients, according to their genetic merit, constitutes a fundamental aspect in ovine AI [11]. Breed characteristics, fertility traits, and the presence of genetic disorders in animals exert a significant impact [12]. Moreover, it is crucial to consider other exogenous factors such as nutrition and prevailing climatic conditions to optimize the success of ovine AI [13]. The interaction between endogenous and exogenous factors emphasizes the complexity of ovine AI and underscores the need for a comprehensive approach to achieve optimal results for such programs. In this paper, we aimed to offer a detailed exploration of how these factors collectively influence the outcomes of ovine AI to provide valuable insights to researchers, veterinarians, and sheep breeders by synthesizing existing knowledge and pinpointing opportunities for improving the efficiency and the success of ovine AI.

## 2. Endogenous Factors: Interplay with AI-Associated Exogenous Factors and Success

**Figure 1:**
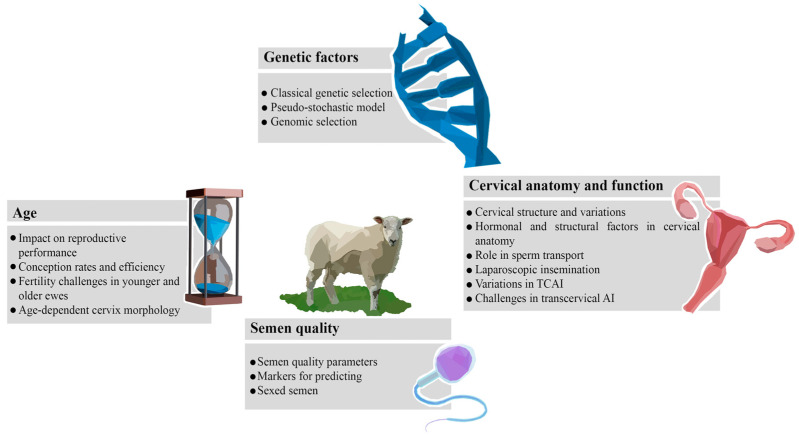
Interaction between endogenous and exogenous factors and their impact on the success of ovine AI. Genetic factors: [17,18]; age: [19,20,21,22]; cervical anatomy and function: [23,24,25]; semen quality: [26]. TCAI: transcervical artificial insemination; and AI: artificial insemination.

The success of ovine AI is influenced by various endogenous factors (Figure 1). Genetic factors play a crucial role, affecting reproductive performance and fertility traits in both rams and ewes [14]. Breeds exhibit distinct reproductive characteristics, impacting the efficiency of AI programs [12]. Age is another determinant, with younger animals often displaying better reproductive outcomes [15]. Additionally, the cervical anatomy of ewes is a critical factor, influencing the ease of semen deposition and subsequent fertilization [16]. Understanding and considering these endogenous factors that are discussed in detail below are essential for optimizing the success of AI in sheep, contributing to enhanced reproductive efficiency and genetic progress in breeding programs.

### 2.1. Genetic Factors

Understanding the nuanced role of genetic factors in the success of ovine AI is crucial. Research analyzing 5,678,168 ovine AI records from French AI centers revealed complex heritability dynamics [17]. While male fertility exhibited low heritability ranging from 0.001 to 0.005, female fertility showed relatively higher estimates between 0.040 and 0.078 [17]. These findings underscore the complexity of improving AI outcomes through classical genetic selection. Nevertheless, advances in reproductive techniques and the integration of genomic information in a single-step approach result in genetic gains. In Assaf and Manchega breeds, it was reported that, despite a low heritability of 7 to 10% and 5%, respectively, a positive trend in breeding values over the last decade has been observed [18]. A pseudo-stochastic model study, evaluating the impact of the proportion of artificially inseminated ewes on genetic gain and inbreeding, indicated that incorporating AI, particularly with progeny testing of AI males, could yield substantial additional genetic gains ranging from +15% to +84% compared to conventional natural mating breeding programs [18]. The model highlighted the importance of AI in advancing genetic progress and the possible repercussion of restricting AI on the breeding population and trait selection. Research on the synergies between genomic selection (GS) and female reproductive technologies, specifically multiple ovulation and embryo transfer (MOET), in sheep breeding programs revealed that, when MOET was added to AI or natural breeding (AI/N) without the use of GS, an extra 25 to 60% genetic gain was observed, whereas, when GS was applied in conjunction with MOET, the rates of genetic gain increased even more significantly, ranging from 38 to 76% [11], especially for breeding programs which focus on the selection of traits measured late in life or which are sex-limited. These results highlight the substantial improvements in genetic gain that can be achieved by strategically combining GS and reproductive technologies, particularly under the principles of optimal contribution selection to control inbreeding. To further promote genetic improvement in sheep via AI, targeted efforts could include refining breeding strategies, increasing the utilization of genomic information, and optimizing AI protocols to maximize the positive impact of genetic factors on overall flock performance and adaptability.

### 2.2. Age

The impact of age as an endogenous factor on ovine AI is significant. Ewes exhibit variations in reproductive performance throughout their lifespan, and age plays a crucial role in influencing fertility outcomes. Generally, ewes at the peak of their reproductive years, typically between 2 and 6 years old, tend to have higher conception rates and reproductive efficiency during ovine AI [19,20,21,22]. Younger ewes may face challenges in reaching optimal reproductive maturity, while older ewes may experience a decline in fertility. In a study conducted by Naqvi et al. [21], Malpura and Avikalin ewes, inseminated by Garole rams of the same age and under identical management practices, demonstrated that the lambing rate exhibited a notable decrease in ewes younger than 2 years and in those older than 6 years [21]. Similarly, in a Churra breed, it was demonstrated that the lambing rate was higher in ewes aged between 1.5 and 4.5 years [15]. The same study showed that fertility also decreased depending on the number of previous parturitions [15]. Regarding younger females, it was suggested that low fertility is potentially attributed to nutritional challenges linked to the high demands for growth and lactation [15]. Moreover, the decline in fertility associated with age and the number of parturitions could be linked to several factors. Firstly, it may be connected to a reduced response of ewes to synchronization due to prior hormonal treatments with equine chorionic gonadotropin (eCG) to induce ovulation and the subsequent development of anti-eCG antibodies. Anti-eCG antibodies are not age-related; rather, they are exogenous factors that can develop over time due to repeated hormonal treatments. These antibodies can interfere with the synchronization process and impact fertility [23,27,28,29]. Additionally, this decrease in fertility could be attributed to a decline in the quality of female gametes or a disruption in the luteal phase [28]. In this context, a recent study realized on Rembi ewes aged between 1.5 and 6 years that were synchronized for the first time showed that ewes aged 3 years and over, inseminated with semen from adult rams, exhibited higher fertility rates [29]. To sum up, it is advisable to form insemination groups comprising ewes aged from 2 to 5 years, while younger and older ewes are better suited for natural mating. The recommendation for natural mating for younger and older ewes is based on the understanding that these age groups may face challenges in responding to synchronization protocols, and their reproductive efficiency could be better supported through natural mating. Additionally, careful considerations should be given to both the number of parturitions and the frequency of hormonal synchronization in ewes.

In addition, the importance of high-quality semen, characterized by good motility, concentration, viability, and normal morphology, cannot be overstated for successful ovine AI [10]. Consequently, research has been directed towards understanding the impact of aging on ram semen quality. Ntemka et al. [30] demonstrated that Chios rams, even up to the age of 13 years, maintain a superior semen quality across various parameters, including motility, kinetics, viability membrane integrity, morphology, chromatin integrity, and sperm membrane biochemical functionality. Similarly, a study on Rembi rams revealed that adult rams (5 to 6 years) exhibited a higher mass motility and concentration compared to their younger counterparts (18–24 months) [31]. However, contradictory findings were observed in Boujaad rams, where the quality parameters of semen and the biochemical composition of seminal plasma were found to be better in younger (2.5 to 3 years) rams than in older (5.5 to 6 years) rams [32]. The inconsistent findings across breeds may be attributed to variations in breed-specific genetic traits, environmental conditions, and management practices.

### 2.3. Cervical Anatomy and Function

In domestic animals, the cervix acts as the gateway for sperm to enter the uterus, and its physical attributes, including length, diameter, and mucus production, are crucial factors influencing the ability of sperm to traverse this barrier [23]. Additionally, the capability of the cervix to relax and contract in response to hormonal cues and insemination procedures is vital for the efficient transport of sperm to the fertilization site [33]. In sheep, the cervical structure is characterized by an elongated fibrous composition, mainly consisting of cartilaginous tissue. This cervical region features four to seven intricate folds, presenting a non-uniform lumen configuration, which are challenging for the passage of an inseminating pipette [24,25]. As a result, the most effective approach for AI in sheep with frozen-thawed semen involves laparoscopic insemination [34,35,36]. This specialized procedure entails the precise deposition of semen directly into the uterine horns, bypassing the complex cervical anatomy [16,24,25]. However, the laparoscopic insemination technique requires a high level of expertise and dedicated resources, making it a preferred choice for initiatives focused on enhancing genetic traits in high-value sheep breeds [37].

The structural complexity of the ewe cervix demonstrates significant variations dependent on breed, age, parity, and physiological state and may explain the varying success rates of transcervical artificial insemination (TCAI). Kaabi et al. [24] reported that these variations generally pose challenges for deep insemination using conventional artificial insemination catheters. El Khalil et al. [16] reported that the cervix morphology in Boujaâd ewes was found to be more complex than in D’man ewes, with this complexity being age-dependent. Precisely, the study concludes that the cervix of D’man ewes is less complex and more favorable for transcervical artificial insemination, while, in Boujaâd sheep, selecting 4-year-old ewes may overcome cervix complexity, enabling successful artificial insemination [16]. Similarly, in Sanjabi ewes, significant age-related differences in cervical anatomy were observed, impacting the success of reproductive biotechnologies [38]. Advancing in ewes’ age highlights a noticeable shift towards larger and less intricate cervix structures with fewer folds. The study emphasizes that cervical penetration is more profound in ewes featuring simpler cervix structures (grade 1) compared to those with more complex configurations (grade 3) [38]. The age-related changes in the cervix of sheep are characterized by variations in cervical anatomy, particularly in the convoluted nature of the cervical lumen. As sheep age, cervices with a less convoluted lumen are found to be more easily penetrated during artificial insemination. Moreover, a notable difference in hormonal concentrations exists between non-luteal and luteal cervices. Specifically, non-luteal cervices, more prevalent in older ewes, exhibit higher concentrations of estradiol [39]. This hormonal distinction induces cervical relaxation, contributing to a more facilitated and deeper penetration during artificial insemination procedures. Regarding age-related variations in cervical types, it is suggested that these changes are linked to a morphological alteration occurring at parturition [39]. The age-related changes in sheep cervix morphology could differ from those observed in other species, emphasizing the need for species-specific considerations in artificial insemination techniques. Comparative studies with other species could provide insights into unique characteristics and variations in cervix morphology associated with aging.

Moreover, Richardson et al. [25] found that, to traverse the ovine cervix, spermatozoa must contend with an outward flow of cervicovaginal mucus, a non-Newtonian fluid serving protective, lubricative, and transport functions. Despite no observed effect of ewe breed on mucus pH or ferning pattern, Belclare ewes exhibited a higher in vitro penetration of cervicovaginal mucus by spermatozoa compared to Suffolk ewes [25]. The properties of cervicovaginal mucus also vary over the estrus cycle, with less hydration and increased viscosity during the luteal phase. These complexities highlight the challenges faced by sperm in navigating the cervix during artificial insemination, emphasizing the importance of understanding both hormonal and structural factors in optimizing insemination success.

### 2.4. Semen Quality

Semen quality plays a critical role in the success of ovine AI programs, impacting fertility, conception rates, and overall production while facilitating genetic enhancements in the flock. However, the assessment of semen quality extends beyond conventional parameters such as progressive motility, morphology, and membrane integrity. To gain a comprehensive understanding of sperm functionality, it is essential to consider additional aspects. Fertilization is a complex process reliant on various sperm capacities, and, thus, evaluating both semen quality and sperm functionality using diverse techniques can significantly enhance the accuracy of predicting fertility rates. Papadopoulos et al. [40] found that conventional in vitro fertilization procedures may not be a reliable predictor of pregnancy rates in field conditions. This suggests a need for potential modifications to better assess differences in sperm fertilizing ability between batches [40]. In a recent study focused on assessing seminal markers for predicting the fertilizing ability of refrigerated seminal doses in ovine AI, it was discovered that specific markers, such as intact membrane, non-capacitated sperm, and DNA-intact spermatozoa, strongly correlate with in vivo fertility and fecundity in artificial insemination [41]. These markers offer valuable tools to optimize AI programs, ultimately leading to improved reproductive outcomes, economic benefits, and increased fertility rates in sheep breeding programs [41]. Other studies also reported a correlation between capacitation status and in vivo fertility in studies conducted with frozen-thawed sperm in cattle [42,43]. To improve artificial insemination in sheep, it is critical to move beyond traditional semen quality measurements and use a more holistic approach. Moreover, recognizing the limitations of traditional in vitro fertilization technologies, it is critical to investigate adaptations that match real-world field situations. Additionally, further studies on seminal markers predictive of fertilizing capacity could help optimize AI programs, promising greater reproductive outcomes and higher fertility rates in sheep breeding.

Considering the critical role of semen quality in ovine AI programs and the importance of additional parameters, it is also important to address the role of sexed semen. With its ability to pre-determine the gender of the offspring, sexed semen could introduce an additional layer of precision to breeding [26]. While this technology has been extensively explored in other livestock [44], its application and impact on fertility rates in sheep warrant further investigation. Integrating the evaluation of sexed semen alongside traditional semen quality measurements and seminal markers predictive of fertilizing capacity could offer a more holistic approach for improving AI programs in sheep, contributing to enhanced reproductive outcomes and increased fertility rates.

## 3. Exogenous Factors

**Figure 2:**
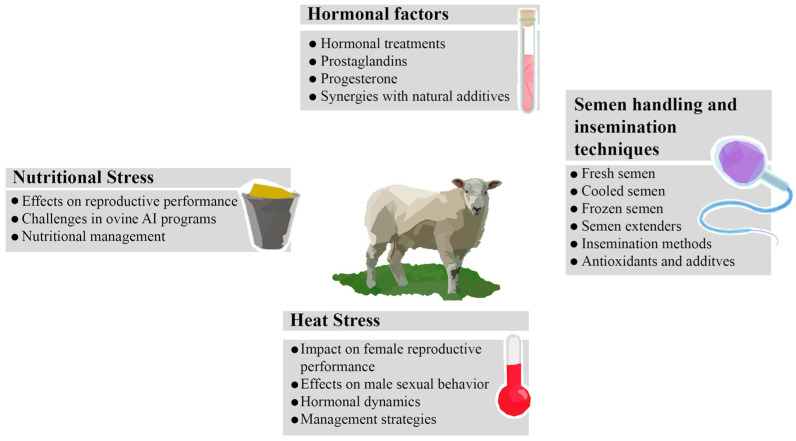
Exogenous factors influencing ovine artificial insemination success. Hormonal factors: [45,46,47]; semen handling and insemination techniques: [48,49]; heat stress: [50]; nutritional stress: [51,52,53].

Several external factors could influence the effectiveness of ovine AI (Figure 2). These include choices between natural and hormonal estrus, insemination techniques, and timing of insemination, as well as the influence of environmental conditions (heat stress and nutritional stress).

### 3.1. Hormonal Estrus

The literature reveals a debate between natural and hormonal methods for estrus induction and synchronization in sheep. Natural estrus is influenced by photoperiod and seasonal variations related to latitudes and is cost-effective [54]. However, its reliance on external factors such as day length and temperature can lead to irregularities in synchronization [43]. Additionally, the use of teaser rams, as an aspect of the ram effect induced by introducing a male ram, stimulates a surge in luteinizing hormone (LH), initiating estrus in ewes via pheromone-triggered neuroendocrine pathways [55,56]. This natural method can circumvent the inhibitory effects of progesterone, prompting estrus even in anestrous ewes, although individual responses may vary [57]. Combining the ram effect with photoperiodic treatments has exhibited efficacy in eliciting estrus outside the typical breeding season, aligning with the inherent reproductive cycles of sheep [58,59]. Nevertheless, challenges persist, such as the duration required for treatment effectiveness and logistical complexities in implementing precise photoperiodic schedules [60]. Therefore, the use of hormonal treatments allows for precise timing and synchronization irrespective of the season, making them favorable in controlled breeding programs [61,62]. Hormonal treatments used to induce synchronization in ewes vary depending on the breeding season (Table 1).

Prostaglandins are a common choice for synchronization during the breeding season, when ewes typically have a functional corpus luteum, making them responsive to this approach [45]. However, it was demonstrated that the use of prostaglandins is time-sensitive due to their role in accelerating luteal dissolution [46,63]. Consequently, they cannot be effectively employed during seasonal anestrus [61]. Hence, outside of the breeding season, progesterone (P4) plays a pivotal role in most synchronization protocols for ewes [47]. It is administered through various methods, including the controlled internal drug-releasing (CIDR) device, medroxyprogesterone acetate (MAP), and fluorogestone acetates (FGA) to facilitate estrous synchronization [47]. Additionally, to enhance fertility, it is common practice to supplement P4-based protocols with the administration of gonadotropins like eCG, pregnant mare serum gonadotropin (PMSG), and gonadotropin-releasing hormone (GnRH) towards the end of the synchronization process [45,47,63]. The typical dosage of MAP often employed for estrous synchronization is 60 mg, although it is worth noting that doses below 40 mg can also yield effective results [46,64]. The standard duration of MAP administration usually spans around 14 days. Similarly, for FGA, the most common dose is 40 mg, and a dose of 30 mg has demonstrated a comparable effectiveness [65,66]. Outside of the breeding season, the FGA treatment period typically ranges from 7 to 14 days, with a shorter duration of 7 days proving sufficient for achieving estrous synchronization [61]. Interestingly, when comparing between MAP, FGA, and CIDR, it appears that 60 mg MAP sponges and CIDR containing 300 mg of progesterone were more suitable choice for estrous synchronization [71,72]. Regarding PMSG, it is typically administered in a range of 200 to 700 IU, with variations based on specific synchronization protocols [65,67,68]. For eCG, the most common dosage falls within the range of approximately 300 to 600 IU [64,69], while GnRH is typically administered at a dosage of about 25 µg [70]. These dosages serve as general guidelines for these hormonal treatments, but it is essential to note that specific protocols and individual circumstances may influence the precise dosage used.

On the other hand, it is important to recognize that hormonal treatments in sheep come with several constraints. These treatments are often deemed expensive, particularly when evaluating the results obtained through AI. Furthermore, extensive research has highlighted potential drawbacks in certain breeds, including the possibility of hormonal treatments causing changes in cervical mucus production, leading to an elevated risk of uterine infections [73,74]. In this regard, a study on six European ewe breeds including Suffolk and Belclare in Ireland, Fur and Norwegian White Sheep (NWS) in Norway, and Ile de France and Romanov in France reported that the use of exogenous hormones (14-day progesterone vaginal sponge and the administration of 400 IU eCG at sponge removal) for synchronization produced differences in mucus production between ewe breeds, although these differences did not affect mucus viscosity [37]. Another study reported an increase in mucus production in multiparous Rambouillet ewes at estrus using sponges impregnated with MAP or FGA [75]. Contrary, Smith and Allison [76] found decreased levels of mucus produced by Merino ewes following progesterone synchronization. Another study realized on Merino ewes reported that prostaglandin synchronized (PGF2α) used for controlled breeding resulted in mucus production like in the natural cycle at the follicular phase [77]. The variation in results in these studies can be attributed to variations in hormonal treatments, breed-specific responses, the age and parity of ewes, the stage of the estrous cycle, and differences in study design. The aforementioned studies provide valuable insights into hormonal treatments for synchronization in sheep, offering a comprehensive overview of protocols and dosages. They include the incorporation of diverse hormonal treatments, dosages, and synchronization protocols, contributing to a holistic understanding. However, a critical appraisal reveals certain limitations. The weaknesses emerge from variations in study design, breed-specific responses, and limited consideration of individual circumstances influencing dosage choices. For instance, studies on mucus production in response to hormonal treatments exhibit inconsistencies, possibly attributed to breed differences, ewe characteristics, and variations in synchronization methods. While these studies significantly contribute to our knowledge, further research addressing these limitations is essential to enhance the reliability and applicability of hormonal synchronization protocols in diverse sheep populations.

While hormonal synchronization methods offer precise control, recent studies have emphasized the potential benefits of combining natural additives, particularly royal jelly (RJ), with these protocols in wool ewes [78,79,80]. However, despite the fact that promising outcomes have been observed, the current understanding of the effects of RJ remains limited and occasionally conflicting. Further well-designed studies are essential to confirm the potential benefits of RJ, ascertain optimal dosages and administration methods, and address any inconsistencies. Additionally, considering the cost implications of RJ and assessing its economic viability for widespread use in large-scale sheep farming are crucial for practical implementation.

### 3.2. Semen Handling and Insemination Techniques

Effective semen handling is a critical aspect for sheep reproduction programs, particularly considering the unique challenges specific to each phase of AI. Prior to AI, stress factors such as temperature fluctuations, mechanical agitation during transportation, and exposure to reactive oxygen species (ROS) can significantly impact the viability and quality of semen [48]. This underscores the importance of meticulous handling during collection, dilution, and cooling processes. Addressing these stress factors is crucial for ensuring optimal semen quality and, consequently, achieving favorable fertility outcomes in ovine AI programs. Therefore, the nuanced reproductive physiology of sheep demands careful consideration of stressors at every stage of semen handling and artificial insemination to maximize the efficacy of breeding strategies and genetic improvement programs, whether employing fresh, cooled, or frozen thawed semen [49]. Semen quality and viability significantly impact the success of ovine AI. The choice of insemination techniques depends on the type of semen used. For fresh or cooled semen, non-surgical cervical or vaginal insemination is typically employed [10]. In contrast, when dealing with frozen semen, especially considering the challenges related to cervical anatomy, laparoscopic insemination becomes the preferred method. Concerning fresh semen, timely use is imperative, as its viability rapidly declines after collection [10]. To maintain semen quality, it can be kept at a controlled temperature (28–30 °C) during insemination. Insemination is carried out on synchronized estrus, and the results obtained range between 56.7% and 70% [81,82,83,84]. Additionally, preserved semen has become an important tool for genetic improvement and has streamlined semen transport. Therefore, the dilution and cooling of semen play crucial roles in inseminating multiple females and providing essential nutrients and protection against pH changes [85]. Extensive studies have investigated this topic using several extenders. However, this process leads to a gradual decline in semen quality, exacerbated by an initial cold shock and sperm dilution, weakening the antioxidant system of seminal plasma [86]. This decline is primarily due to the production of ROS during the metabolism and the sensitivity of ram sperm membranes to oxidative stress [87]. Consequently, sperm motility, viability, membrane integrity, and fertilization ability decrease. To counteract this, studies have explored the addition of exogenous antioxidants to semen extenders to maintain cell function and balance ROS and antioxidants [48]. In recent years, various biomolecules derived from plants such as *Artemisia incana*, *Camellia sinensis*, *Echinacea purpurea*, *Entada abyssinica*, *Foeniculum vulgare*, *Moringa oleifera*, *Nigella sativa*, *Punica granatum*, *Rosmarinus officinalis*, *Syzygium aromaticum*, *Thymus vulgaris*, and *Zingiber officinale* have emerged as cost-effective and natural additives to improve sperm function during storage [87,88]. A recent study highlighted the beneficial effects of purified c-phycocyanin from *Spirulina platensis* on the quality of stored Boujaâd ram semen and its in vivo fertility [89]. Cueto and Gibbons [90] reported that preserving semen at 15 °C is suitable for short-term storage (around 8–12 h), whereas preserving semen at 5 °C is more appropriate for longer periods (approximately 12–24 h). Furthermore, factors like semen concentration and the choice of semen extender also play a crucial role in influencing fertility rates (Table 2). For instance, a study realized by Naim et al. [91] demonstrated that inseminating with 300 million sperm stored at 5 °C led to higher fertility rates in Merino ewes compared to using 150 million sperm. In the same breed, Olivera et al. [92] found that using semen extended in Piedra Mora (consisting of UHT skim milk, egg yolk, and glycerol) resulted in a higher fertility rate when contrasted with semen extended in TRIS (comprising TRIS, fructose, citric acid, and egg yolk). In the Sardi breed, the highest fertility rate, reaching 73%, was achieved when using semen extended in skim milk and stored at 15 °C for 4 h. In comparison, semen extended in skim milk and stored at 5 °C for the same duration resulted in a slightly lower fertility rate of 61% [84]. Additionally, when Duragen^®^ extender was used as an alternative to skim milk, it yielded promising results, with a 66% fertility rate [93].

Frozen semen plays a pivotal role in animal breeding and AI. Sperm is carefully preserved at approximately −196 °C in liquid nitrogen, facilitating extended storage and convenient transportation. This technique allows the conservation of genetic material from high-quality males, ensuring its viability even beyond their active reproductive period [87]. Thawed frozen semen is then used for efficient insemination to promote desirable genetic traits in animal populations. In sheep, the use of cervical or vaginal AI using frozen-thawed semen has yielded low fertility rates, such as 4% [34], 28% [94], and 13% [95]. This low fertility can be attributed to the anatomical characteristics of the ovine cervix, as cited above. Consequently, the most effective approach for frozen-thawed semen AI in sheep obliges the use of laparoscopic insemination, a surgical procedure which demands veterinary expertise [96]. Hence, this method is primarily applied in genetic improvement programs involving high-value animals. However, it should be highlighted that there are some exceptions, as reported by Paulenz et al. [97], who achieved noteworthy fertility outcomes when employing frozen-thawed semen in the Norwegian White Sheep (NWS) breed. This efficacy is linked to the distinctive ability of frozen-thawed sperm to navigate the cervix, a trait observed in certain ewe breeds and not others [98].

### 3.3. Heat Stress

Several studies have indicated that certain sheep breeds, such as Ouled Djellal (Algeria), Dorper (Thailand), Hamari (Sudan), Suffolk (Egypt), Persian Karakul (Iran), Friesian, and Chios (Greece) rams, exhibit a better semen quality during summer and spring compared to other seasons [99,100,101,102,103,104]. In arid regions of Algeria, Rembi rams demonstrated reduced libido during the scorching summer months when temperatures reached or exceeded 39.5 °C [29]. Furthermore, HS can influence sexual behavior. It has been observed to diminish various aspects of male sexual performance, including reaction times for ejaculation, total time taken for ejaculation, latency periods, and the number of mounts during ejaculation [105,106,107]. These findings collectively suggest that heat stress could potentially influence the success of artificial insemination in sheep, emphasizing the need for careful management practices during adverse climatic conditions.

Similarly, HS affects ewes, leading to reduced sexual behaviors such as circling, tail-fanning, head-turning, standing, and approaching the ram in breeds like Bharat Merino [108]. Additionally, HS can have a detrimental effect on oocyte and embryo quality, particularly when ewes are exposed to hyperthermia within the first three days after AI. This can result in reduced fertility rates and early embryo mortality, as reported by a study where 69.2% of cleaved ovules were observed after ewes were exposed to hyperthermia on the twelfth day of their reproductive cycle [109,110]. HS also affects the hormonal dynamics of ewes, reducing behavioral estrus and luteinizing hormone surges and plasma progesterone levels between days 7 and 13 of the estrous cycle [111]. Furthermore, it impacts body weight, estrus duration, the birth weight of lambs, and hormonal profiles, particularly estradiol 17-β and progesterone, as observed in Malpura ewes subjected to hyperthermia and restricted feeding [112]. These findings underscore the possible multifaceted effects of heat stress on AI success and reproduction in sheep. Recently, it has been shown that HS diminishes embryo production during AI or embryo transfer procedures in Australian Merino sheep [111].

To address the deleterious impact of HS on ovine AI, it is necessary to adopt a multi-faceted approach. These include the provision of adequate shade, meticulous adjustment of the timing of AI procedures to circumvent heat peaks, selection of heat-tolerant sheep breeds, careful attention to nutrition and hydration, and assiduous surveillance of prevailing environmental conditions. At the same time, raising awareness, educating stakeholders, and adapting strategies to the unique climatic conditions of the region are essential to improve AI success in heat stress situations. Collaboration with experts and research institutes further enriches the repertory of HS management practices.

### 3.4. Nutritional Stress

Nutritional stress (NS) also profoundly impacts the reproductive performance of sheep, particularly those raised in arid and semi-arid regions [109]. In these challenging environments, limited access to quality forage and water can lead to inadequate nutrition, which in turn affects the reproductive efficiency of the flock [113,114]. NS often results in the delayed onset of puberty, extended estrous cycles, and irregular or failed ovulation in ewes [115,116]. Moreover, the reduced body condition and energy reserves in malnourished sheep can lead to lower conception rates and increased embryo mortality [117,118]. NS significantly impacts ram reproductive performance, affecting sexual behavior, scrotal and testicular measurements, sperm characteristics, and semen quality [51,52]. This unfavorable reproductive outcome not only affects natural breeding but also poses challenges to ovine AI programs. These programs are dependent on precise timing and synchronization of estrus cycles, which become challenging when NS disrupts these cycles. Additionally, poor nutrition could compromise the overall health and immune function of sheep, making them more susceptible to reproductive diseases [53,109]. Considering the detrimental effects of NS on sheep reproduction in arid and semi-arid regions, it is necessary to prioritize effective nutritional management strategies. This involves ensuring the availability of high-quality forage and water, conducting regular assessments of sheep body condition, providing timely nutritional supplementation, and optimizing breeding timing to mitigate the impact of limited resources. Additionally, promoting disease control and awareness as well as understanding the challenges associated with ovine AI in such environments can collectively contribute to improved reproductive outcomes.

## 4. Conclusions

In summary, this review underscores the complex interaction between endogenous and exogenous factors influencing the success of ovine AI. As mentioned, understanding genetic dynamics, age-related fertility variations, cervical anatomy complexities, and the crucial role of semen quality highlights the need for careful consideration of these endogenous factors in AI programs. Meanwhile, the multifaceted nature of exogenous factors, involving the debate over natural vs. hormonal synchronization, effective semen handling, and the impact of environmental stressors, accentuates the complexity of optimizing ovine AI outcomes. The recommended strategies, such as incorporating natural additives, refining semen handling protocols, and addressing challenges from heat and nutritional stress, provide practical insights for researchers, veterinarians, and sheep breeders. This review serves as a guide, advocating for a holistic approach which recognizes and harmonizes both internal and external factors to enhance the efficiency and success of ovine AI programs. Implementing these recommendations has the potential to significantly contribute to improved reproductive outcomes and genetic progress in sheep breeding practices.

## Figures and Tables

**Table 1:** The most used hormonal treatment protocols for inducing synchronization in ewes.

Hormonal Treatment	Dosage	Administration Method	Commonly Used in Breeding Season	Commonly Used Outside Breeding Season	References
PG	Varies according to the analog used	Double injection with an 11-day interval	Yes	No	[45,46,63]
P4	MAP: 60 mg FGA: 40 mg CIDR: 300 mg	CIDR, MAP, FGA	No	Yes	[47,64,65,66]
PMSG	200–700 IU	Injectable	Yes	Yes	[65,67,68]
eCG	300–600 IU	Injectable	Yes	Yes	[64,69]
GnRH	25 µg	Injectable	Yes	Yes	[70]

PG: prostaglandins; P4: progesterone; PMSG: pregnant mare serum gonadotropin; eCG: equine chorionic gonadotropin; GnRH: gonadotropin-releasing hormone; MAP: medroxyprogesterone acetate; FGA: fluorogestone acetates; and CIDR: controlled internal drug releasing.

**Table 2:** Synthesis of semen handling and insemination practices in some sheep breeds.

Breed	Storage (°C)	Extender	Duration (h)	Concentration (Sperm × 10^6^)	Fertility (%)	References
Merino	5	OviPro^®^	24	300	29	[91]
				150	14	
	5	TRIS	24	120	19	[92]
		Piedra Mora			49	
		TRIS	48		22	
		Piedra Mora			47	
Sardi	15	SM	4	400	73	[84]
	5				61	
	5	Duragen^®^	4	400	61	
Boujaad	5	SM	4	400	63	[89]
		SM + 2.4 µg/mL of C-PC			76	

TRIS: TRIS, fructose, citric acid, egg yolk; Piedra Mora: UHT skim milk, egg yolk, glycerol; SM: skim milk; and C-PC: phycocyanin purified from *Spirulina platensis*.

## Data Availability

Data are contained within the article.
